# Effects of Artificial Vegetation Restoration Pattern on Soil Phosphorus Fractions in Alpine Desertification Grassland

**DOI:** 10.3390/plants14101429

**Published:** 2025-05-10

**Authors:** Hongyu Qian, Nairui Yang, Haodong Jiang, Yinan Li, Ao Shen, Yufu Hu

**Affiliations:** College of Resources, Sichuan Agricultural University, Chengdu 611130, China; qhy1632021@163.com (H.Q.);

**Keywords:** soil, plant restoration, phosphorus fractions, alpine sandy land, physicochemical properties

## Abstract

Phosphorus (P) is essential for plant growth, but its soil availability depends on the characteristics of P fractions. However, few studies have examined soil P fractions under ecological restoration in alpine and semi-humid regions. This study investigated three restoration methods on the eastern Tibetan Plateau: planting mixed grasses (MG), planting *Salix cupularis* alone (SA), and planting *Salix cupularis* in combination with grasses (SG), restored for 14 years, with untreated sandy land (CK) as control. Through field sampling and laboratory analysis, soil P fractions and physicochemical properties were analyzed. The findings demonstrate that the three ecological restoration modes could increase total P and total organic P content and reduce inorganic P content. Ecological restoration can improve the content of soil labile P (resin-Pi, NaHCO_3_-Pi, and NaHCO_3_-Po) by activating NaOH-Pi and HCl-P, thus improving the availability of soil P and increasing the potential P (residual-P) source. Soil P fractions content positively correlated with SWC, SOC, and TN (*p* < 0.05) but negatively with BD and pH (*p* < 0.05). The experimental outcomes of this study will help to understand the P availability and its potential sources during ecological restoration while providing a scientific foundation for selecting optimal restoration strategies in alpine sandy land.

## 1. Introduction

Phosphorus (P) is an essential nutrient element for plant growth, and its biogeochemical cycle plays a pivotal role in governing ecosystem evolution and stability [1]. Soil P availability, a critical determinant of ecosystem-level P cycling [2], is principally regulated by the content and proportional distribution of soil P fractions [3]. P in soil is classified into inorganic P (Pi) and organic P (Po) fractions [4]. In Hedley’s P classification method modified by Tiessen and Moir, soil P is further categorized into labile P, moderately P, and non-labile P based on bioavailability [5,6]. There is a complex conversion relationship between Po and Pi in soil [7]. Among them, soil Po can be mineralized and decomposed into Pi under the action of enzymes, which is absorbed and utilized by plants, and is an important source of P available to plants [2,8]. Ecological restoration drives shifts in vegetation biomass and diversity, thereby regulating plant-mediated P sequestration and its subsequent translocation to soils. These dynamics consequently alter transfer processes including Po mineralization, Pi adsorption, precipitation, and dissolution, ultimately modifying soil P speciation and bioavailability [9]. Therefore, it is of great significance to study the changes in soil P content and its proportion under ecological restoration to reveal the availability and supply capacity of soil P. Although many studies have shown that ecological restoration can increase the content of soil labile P and moderately P, and improve the availability of soil P [10,11], there were significant differences in soil P form content and its proportion in different ecological restoration models. For instance, Fu et al. [12] demonstrated in their investigation of subtropical montane ecosystems under varying restoration regimes that natural secondary forests exhibited significantly higher contents of water-soluble Pi and Po fractions compared to pine plantations, eucalyptus stands, and shrublands. Similarly, Chen et al. [13], in their examination of three vegetation restoration strategies in the subalpine zone of western Sichuan, documented elevated concentrations of citrate-extractable P and enzyme P under natural regeneration regimes relative to anthropogenic restoration practices, whereas HCl-extractable P exhibited preferential accumulation in intensively managed plantation systems. Therefore, it is necessary to pay more attention to the difference in soil P fraction characteristics in different ecological restoration models.

The alpine grassland on the eastern edge of the Tibetan Plateau is located in the alpine semi-humid area. It is the world’s largest plateau peat swamp wetland and an important water conservation area of the Yangtze and Yellow Rivers [14,15]. However, under the influence of natural and human factors such as global climate change, wind erosion, rat damage, ditching and drainage, and overgrazing for a long time, the alpine grassland in this region has been seriously degraded, and the local desertification problem is prominent, which poses a serious threat to regional ecological environment security and sustainable development of animal husbandry production [16,17]. In recent years, in order to curb the further expansion of desertification, a series of ecological restoration work has been carried out on the eastern edge of the Tibetan Plateau, and a typical ecological restoration model has been formed, which mainly consists of planting mixed grasses (MG), planting shrub with *Salix cupularis* alone (SA), and planting shrub with *Salix cupularis* in combination with grasses (SG) [18,19,20]. However, current studies have few reports on the changes in soil P fractions under ecological restoration in alpine areas.

Therefore, in this study, three typical alpine sandy land ecological restoration models (MG, SA, and SG) were taken as the object, and the completely sandy bare land was taken as the control to explore the soil P content and proportion characteristics of the subhumid sandy land in the eastern margin of the Qinghai–Tibet Plateau under different restoration models, and to analyze the correlation between soil P fractions and soil physicochemical properties. To reveal the availability and supply capacity of soil P under ecological restoration. This study postulates three principal hypotheses: (1) Vegetation restoration enhances total soil P stock and augments its bioavailability through improved biogeochemical cycling. (2) Restoration-induced modifications in soil physicochemical properties mediate the alterations in P fractions dynamics across soil profiles. (3) Differential responses of soil P fractions emerge among restoration modalities, with SG exhibiting the most pronounced modifications in P speciation architecture.

## 2. Results

### 2.1. Characteristics of TPi and TPo in Soil

Compared with CK, the three ecological restoration models significantly increased soil TPo content in different soil depths, ranging from 25.33 to 137.07 mg∙kg^−1^, with the largest increases in the 20–40 cm soil layer in the MG restoration model and 40–60 cm soil layer in SG mode. TPi in the 0–20 cm soil layer of the SA restoration model increased significantly (54.92–91.02 mg∙kg^−1^), while TPi in the 20–40 cm and 40–60 cm soil layer of the MG and SG modes decreased significantly. The decrease was greatest in the MG restoration model 20–40 cm soil layer and the SG mode 40–60 cm soil layer (Table 1). In general, ecological restoration can reduce the Pi content in soil and accumulate in the form of Po, and the effect is best in the MG mode 20–40 cm soil layer and the SG mode 40–60 cm soil layer.

### 2.2. Characteristics of P Fractions Content

**Figure 1:**
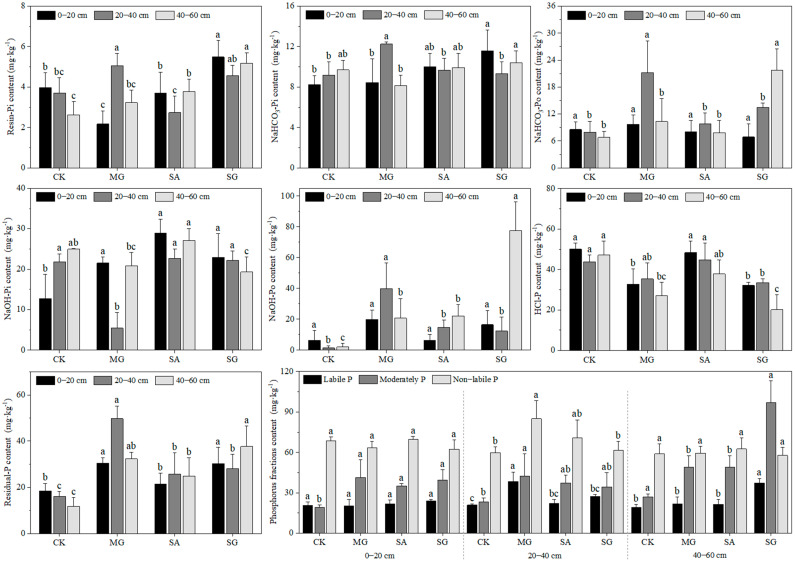
Soil phosphorus content characteristics under different ecological restoration modes. Note: Different letters above each bar indicate significant differences among different restoration years at *p* < 0.05. Vertical bars denote the standard error of the means (*n* = 4).

The results showed that the content of labile P fractions was 19.08–38.46 mg∙kg^−1^ in the three ecological restoration models. Compared with CK, the content of labile P in soil layers of 20–40 cm in MG and 20–60 cm in SG significantly increased (Figure 1). The content of soil resin-Pi in the 0–20 cm soil layer significantly decreased by 1.80 mg∙kg^−1^ in MG mode and increased by 1.51 mg∙kg^−1^ in SG mode. The MG mode only in the 20–40 cm soil layer significantly increased by 1.35 mg∙kg^−1^. The SA and SG modes in the 40–60 cm soil layer significantly increased by 1.13 and 2.55 mg∙kg^−1^, respectively. NaHCO_3_-Pi content was significantly increased by 3.36 mg∙kg^−1^ and 3.05 mg∙kg^−1^ only in the 0–20 cm soil layer of the SG mode and the 20–40 cm soil layer of the MG mode. NaHCO_3_-Po content in the MG mode 20–40 cm soil layer and the SG model 40–60 cm soil layer was significantly increased by 13.20 and 14.96 mg∙kg^−1^.

In the three ecological restoration modes, the content of moderately P fractions was 18.97–96.92 mg∙kg^−1^. Compared with CK, the three modes in the 0–20 cm soil layer significantly increased, only the MG mode in the 20–40 cm soil layer significantly increased, and the SA and SG modes in the 40–60 cm soil layer significantly increased (Figure 1). The NaOH-Pi content in the 0–20 cm soil layer was significantly increased by 8.81, 16.26, and 10.19 mg∙kg^−1^ under the MG, SA, and SG modes, respectively. The MG mode in the 20–40 cm soil layer significantly decreased by 16.47 mg∙kg^−1^. The SG mode in the 40–60 cm soil layer was significantly reduced by 5.64 mg∙kg^−1^. There was no significant difference in NaOH-Po content among the three ecological restoration modes in the 0–20 cm soil layer. The MG model in the 20–40 cm soil layer significantly increased by 38.62 mg∙kg^−1^. In the 40–60 cm soil layer, the MG, SA, and SG models significantly increased by 18.65, 20.04, and 75.66 mg∙kg^−1^, respectively.

In the three ecological restoration modes, the content of non-labile P was 57.78–84.94 mg∙kg^−1^. Compared with CK, only the 0–20 cm soil layer increased significantly under MG mode, and there was no significant difference between the soil layer and the model and the soil layer (Figure 1). Among them, HCl-P significantly decreased by 17.28 mg∙kg^−1^ and 18.01 mg∙kg^−1^ under the MG and SG modes in the 0–20 cm soil layer, respectively. The SG mode of the 20–40 cm soil layer significantly decreased by 10.29 mg∙kg^−1^. In the 40–60 cm soil layer, the MG and SG modes significantly decreased by 20.09 and 27.05 mg∙kg^−1^, respectively. Residual-P content in soil was significantly increased by 11.99 mg∙kg^−1^ and 11.91 mg∙kg^−1^ under the Mg and SG modes in the 0–20 cm soil layer, respectively. In the 20–40 cm soil layer, the MG, SA, and SG modes significantly increased by 33.65, 9.68, and 12.08 mg∙kg^−1^, respectively. In the 40–60 cm soil layer, the MG, SA, and SG modes significantly increased by 20.58, 12.99, and 25.95 mg∙kg^−1^, respectively. In general, under the three ecological restoration, soil P mainly existed in the form of non-labile P, and the content of HCl-P was the highest.

### 2.3. Characteristics of the Ratio of Soil P Fraction to TP

**Figure 2:**
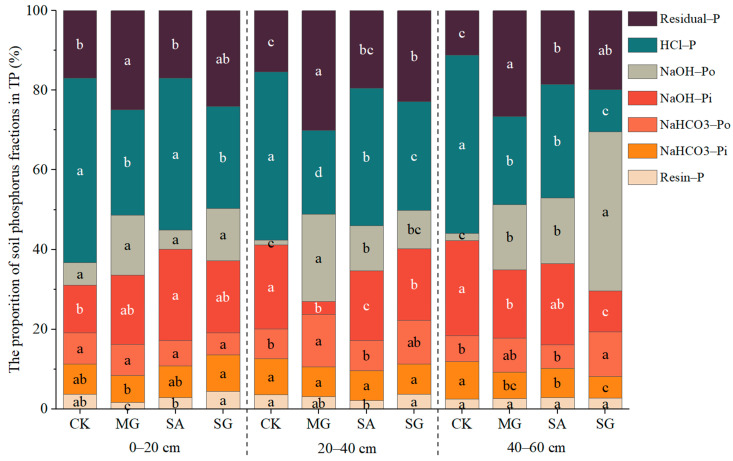
The proportion of P fractions to TP in soil under different ecological restoration modes. Note: Different letters above each bar indicate significant differences among different restoration years at *p* < 0.05. Vertical bars denote the standard error of the means (*n* = 4).

In general, HCl-P accounted for the largest proportion of TP, while resin-Pi accounted for the smallest proportion under different ecological restoration modes. The three ecological restoration models decreased the proportions of NaHCO_3_-Pi and NaOH-Pi and the proportions of 0–60 cm HCl-P in 0–20 cm and 20–40 cm soil layers and increased the proportions of 0–60 cm NaOH-Po and residual-P in 0–60 cm soil layers (Figure 2). Compared with CK, the ratio of resin-Pi in the 0–20 cm soil layer was significantly reduced by 1.97% in MG mode. The NaOH-Pi ratio was significantly increased by 11.06% in SA mode. The proportion of HCl-P in the MG and SG modes decreased by 19.99% and 20.78%, respectively. The residual-P ratio significantly increased by 8.01% under MG mode. The proportions of NaHCO_3_-Pi, NaHCO_3_-Po, and NaOH-Po were not significantly different from CK under the three ecological restoration modes. The ratio of resin-Pi in the 20–40 cm soil layer was significantly reduced by 1.46% under SA mode. The ratio of NaHCO_3_-Pi was not significantly different from CK under the three modes. NaHCO_3_-Po was significantly increased by 5.60% in MG mode. The NaOH-Pi ratio decreased by 17.78%, 3.46%, and 3.04% under the MG, SA, and SG modes. The proportion of NaOH-Po in the MG and SA modes was significantly increased by 20.70% and 10.04%, respectively. The proportion of HCl-P in the MG, SA, and SG modes decreased by 21.07%, 7.57%, and 14.85%, respectively. The residual-P ratio significantly increased by 14.57% and 7.42% in the MG and SG modes, respectively. In the 40–60 cm soil layer, there was no significant difference between the resin-Pi ratio and the CK ratio under three ecological restoration modes. The proportion of NaHCO_3_-Pi in the MG, SA, and SG modes decreased by 2.64%, 1.86%, and 3.83%, respectively. NaHCO_3_-Po significantly increased by 4.70% in SG mode. The NaOH-Pi ratio was significantly reduced by 6.72% and 13.74% under the MG and SG modes. The proportion of NaOH-Po in the MG, SA, and SG modes was significantly increased by 14.59%, 14.68%, and 38.31%, respectively. The proportion of HCl-P in the MG, SA, and SG modes decreased by 22.67%, 16.29%, and 34.22%, respectively. The residual-P ratio significantly increased by 15.39%, 7.38%, and 8.60% in the MG, SA, and SG modes, respectively.

### 2.4. Correlation Between Soil P Fractions and Physicochemical Properties

The results of correlation analysis showed that the content of soil P fractions was significantly correlated with the basic physical and chemical properties of soil (Table 2). SWC was positively correlated with TP, TPo, NaHCO_3_-Po, and NaOH-Po, and negatively correlated with TPi and HCl-P (*p* < 0.05). BD was significantly negatively correlated with TP, TPo, NaHCO_3_-Po, NaOH-Po, and residual-P, and significantly positively correlated with TP, TPi, NaOH-Pi, and HCl-P (*p* < 0.05). The pH was significantly negatively correlated with TP, TPo, NaHCO_3_-Po, NaOH-Po, and residual-P (*p* < 0.05). SOC was significantly positively correlated with TP, TPo, resin-Pi, NaHCO_3_-Pi, NaHCO_3_-Po, NaOH-Po, and residual-P, and was significantly negatively correlated with TPi, NaOH-Pi, and HCl-P (*p* < 0.05). TN was significantly positively correlated with TP, TPo, NaHCO_3_-Pi, NaHCO_3_-Po, NaOH-Po, and residual-P, and negatively correlated with TPi, NaOH-Pi, and HCl-P (*p* < 0.05).

## 3. Discussion

In the results of this study, long-term ecological restoration significantly increased soil TPo content, while significantly reducing soil TPi content, which aligns with the P dynamics observed by Slazak et al. [7] in pine-oak forests following afforestation. This phenomenon may be attributed to the increased plant consumption of soil Pi and the biological retention of soil P [21,22]. More importantly, it was found that soil TPi and TPo changed significantly in the 20–40 cm soil layer of the MG mode and the 40–60 cm soil layer of the combination of the SG mode than in other models and soil layers, possibly because the organic acids secreted by the root can significantly activate soil P [22]. The MG and SG models have higher subsurface root biomass and different soil layers where the main roots gather [19]. Correlation analysis results showed that soil TPi and TPo had opposite relationships with soil basic physical and chemical properties, and were significantly correlated with SWC, BD, SOC, and pH, which indicated that ecological restoration could regulate the ratio of inorganic P to organic P by increasing SWC, SOC, and TN contents and reducing soil BD. In general, ecological restoration can increase the TPo content and reduce the TPi content in the soil, and the effect is best in the 20–40 cm soil layer of the MG mode and the 40–60 cm soil layer of the SG mode.

The results showed that compared with CK, resin-P and NaHCO_3_-Pi were significantly increased in the 20–40 cm soil layer of MG and the 40–60 cm soil layer of the SG mode, while NaHCO_3_-Pi was significantly increased in the 40–60 cm soil layer of the SA mode. This study demonstrates that the MG and SG modes were more conducive to the accumulation of soil labile Pi, and the distribution was different in different soil layers. However, resin-P in the 0–20 cm soil layer of MG mode decreased significantly compared with CK, which may be due to the rapid consumption of soil labile inorganic P fractions due to larger plant biomass and richer diversity in the MG mode [23]. NaHCO_3_-Po is an organic compound that is easily mineralized by microorganisms in a short period of time and is one of the P fractions with high activity, which can be used as the main source of available P [24]. This study showed that the change of NaHCO_3_-Po content was roughly the same as that of resin-P and NaHCO_3_-Pi, and the increase in NaHCO_3_-Pi in the 20–40 cm soil layer of MG and the 40–60 cm soil layer of SG was larger than that of resin-P and NaHCO_3_-Pi. Moreover, from the perspective of the proportion of soil labile P, the ratio of NaHCO_3_-Po in 20–40 cm and 40–60 cm soil layers was significantly increased by the MG and SG modes, respectively, which indicated that the MG and SG modes can improve the availability of soil P mainly by increasing the content of NaHCO_3_-Po. This phenomenon may be attributed to the significant correlations between NaHCO₃-extractable P and both SOC and TN contents (Table 3). The MG and SG restoration modes substantially enhanced vegetation coverage and biomass production, thereby increasing SOC and TN concentrations. These improvements stimulated microbial anabolism and elevated phosphatase activity [25,26], ultimately enhancing the contribution of NaHCO₃-extractable P to soil P availability.

The moderately P (NaOH-Pi and NaOH-Po) are usually combined with soil iron and aluminum compounds in soil and adsorbed on soil aggregates, which can be used by plants through microbial mineralization [27]. The results of this study showed that in the 0–20 cm soil layer, the three restoration modes significantly increased the NaOH-Pi content, but the NaOH-Po content did not change significantly, indicating that the moderately P in the surface soil was mainly accumulated in the form of the inorganic state during the ecological restoration process of the alpine sandy land on the eastern edge of the Tibetan Plateau. In the 20–40 cm soil layer, the contents of NaOH-Pi and NaOH-Po did not change significantly under the SA and SG modes, but significantly decreased and increased under the MG mode, respectively, which corresponded with the changes in labile P. Therefore, the moderately P in the 20–40 cm soil layer of MG mode was mainly accumulated in organic form, and the main source of labile P was NaOH-Pi. In the 40–60 cm soil layer, NaOH-Po increased significantly under the three recovery modes, which may be due to the continuous accumulation of organic matter caused by biological activities [28]. In SG mode, NaOH-Po content significantly increased and NaOH-Pi content significantly decreased, which may be consistent with the explanation of NaOH-Pi change in MG, indicating that NaOH-Pi is also an important source of soil labile P under the SG. Correlation analysis results showed that SOC was significantly negatively correlated with NaOH-Pi and positively correlated with NaOH-Po. Combined with the above content, it was indicated that soil moderately P was mainly accumulated in the form of Po under ecological restoration, and the main source of soil labile P was NaOH-Pi.

Soil HCl-P is mainly combined with calcium to form insoluble P fractions, the proportion of which can be used to evaluate the relative state of soil development. Generally, the higher the degree of weathering, the lower the content of HCl-P [29]. The results show that, on the whole, the proportion of HCl-P in TP is higher than that of other P fractions, which is consistent with the research results of Guan et al. [30]. This is mainly because the soil in the study area is developed from sandy land, and the perennial low temperature makes the chemical weathering and biological processes of the soil extremely slow, and the soil weathering degree is low [31]. There was no significant change in the HCl-P content in the SA model compared with CK, while the HCl-P content in the surface and deep soil in the MG and SG models significantly decreased. The correlation study results showed that HCl-P was significantly negatively correlated with the SWC, SOC, and TN contents, indicating that the SG model had a strong promotion effect on the soil weathering process. It also shows that HCl-P is an important source of available P in the process of ecological restoration in the alpine sandy land on the eastern edge of the Tibetan Plateau. The residual P is a non-labile P fraction in soil, which is difficult for plants to directly absorb and utilize, and must be transformed into a form easily absorbed and utilized by vegetation through a series of processes such as mineralization and weathering [32]. Zhang et al. [33] found that higher residual-P content plays a crucial role in soil P availability during subtropical forest vegetation succession. We found that ecological restoration significantly increased residual-P content and TP proportion in the 20–60 cm soil layer on the whole. This indicates that ecological restoration improves the availability of soil P, but also increases the storage capacity of soil P. The residual P is very difficult to utilize in a short period of time, but with the growth of plants and the succession of communities in the process of ecological restoration, it is highly likely to become a potential P source in soil.

## 4. Materials and Methods

### 4.1. Site Description

**Figure 3:**
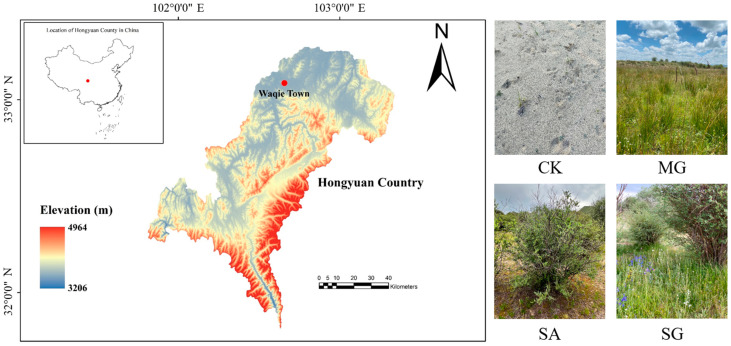
Location of the study area and images of different restoration modes. Note: CK, extremely degraded grassland. MG, planting mixed grasses. SA, planting shrubs with *Salix cupularis* alone. SG, planting shrubs with *Salix cupularis* plus mixed grasses. The same as below.

This research was conducted in Hongyuan Country (33°1′ N, 102°37′ E), Sichuan Province, China, situated along the eastern periphery of the Tibetan Plateau, within a designated demonstration area for degraded grassland restoration [19,20]. The average elevation exceeds 3400 m. This region experiences a continental plateau cold-temperate monsoon climate characterized by short spring and autumn seasons, prolonged winters, and an absence of summer, accompanied by a mean annual precipitation of 791.95 mm concentrated predominantly from May to October. The mean annual temperature is 1.1 °C, with the coldest month average temperature being −10.3 °C, and the warmest month average temperature is 10.9 °C. The average annual snow period is 76 days, without an absolutely frost-free period. The area receives substantial solar irradiation, evidenced by an annual sunshine duration of 2158.7 h and a total solar radiation of 6194 MJ·m^−2^. Dominant soil types comprise meadow soil, swamp soil, and peat soil [19,20]. Owing to persistent anthropogenic pressures including population growth and overgrazing, coupled with natural disturbances such as global warming and rodent/insect infestations, severe degradation of alpine grasslands has occurred in the eastern margin of the Tibetan Plateau, with localized desertification observed in some areas [17,19]. The dominant vegetation species in the recovery area are mainly *Salix cupularis*, *Carex peaeclara*, *Kovresia pygmaea*, and *Elymusnutans*. Since 2007, three typical restoration modes have been piloted in the region to rehabilitate degraded grasslands, with the following specific measures included: planting mixed grasses (MG), planting shrubs with *Salix cupularis* alone (SA), and planting shrubs with *Salix cupularis* plus grasses (SG) [19,20]. In 2021, these restoration modes have been successfully established in the restored area (Figure 3 and Table 3).

In mid-August 2021, three sites for testing ecological restoration patterns were selected on the eastern edge of the Tibetan Plateau, Hongyuan Country alpine sandy land ecological management demonstration area. These included MG, SA, and SG, with the adjacent sandy land (CK) without artificial intervention as the control sample. In the above three test areas, with basically the same site conditions selected, a 25 × 25 m sample plot, and the distance between the sample plot and the boundary in the test area, is more than 100 m. In the two experimental areas of MG and SA, four representative 1 m × 1 m quadrats as repetition. In the two experimental areas of SA and SG, four representative 1 m × 1 m small quadrats are used as repetition (the soil water content (SWC), bulk density (BD), pH, soil organic carbon (SOC), and total nitrogen (TN) content in the samples plots is shown in Jiang et al. [20]).

### 4.2. Measurement Parameters

Soil samples were systematically collected from three depth intervals (0–20 cm, 20–40 cm, and 40–60 cm) within each 1 m × 1 m sampling quadrat. For each soil layer, three replicate subsamples were obtained and thoroughly homogenized before being placed in labeled plastic bags for transportation to the laboratory. Prior to analysis, all collected samples were carefully processed to remove any plant debris, root fragments, and visible soil fauna.

The soil total phosphorus (TP) was determined colorimetrically at 700 nm wavelength using the molybdenum blue method on a Model 721 spectrophotometer after digestion with H_2_SO_4_-HClO_4_ [34]. A modified version of Sui et al. [35] fractionation scheme was used to sequentially extract various forms of Pi and Po soil P. An air-dried soil sample that was <2 mm with a weight of 0.5 g was placed in a 50 mL centrifuge tube and was sequentially extracted with 30 mL each of deionized water, 0.5 mol·L^−1^ NaHCO_3_ (pH = 8.2), 0.1 mol·L^−1^ NaOH, and 1 mol·L^−1^ HCl. The extraction was oscillated for 16 h each time, and then the supernatant was determined by centrifugal stratification. The extraction of P from deionized water and 1 mol·L^−1^ HCl was directly determined by colorimetry (Model 721 spectrophotometer, 700 nm wavelength). P extracted from NaOH and NaHCO_3_ contains two forms of Po and Pi, and the Pi part is directly determined by molybdenum blue colorimetry. The TP content of the extract was determined by the potassium persulfate digestion method, and the Po content was determined from the difference in TP minus Pi. Finally, the residual-P content in the residual soil was determined by high-temperature deboiling with concentrated H_2_SO_4_-H_2_O_2_ and molybdenum antimony resistance colorimetric method.

### 4.3. Statistical Analysis

Data were processed using Microsoft Excel 2021. Statistical analysis was performed using SPSS Statistics 27.0. Graphical representations were generated using Origin 2022 Pro. A one-way ANOVA following Fisher’s least significant difference (LSD) test was used for comparison of index differences (means at *p* < 0.05). Pearson’s analysis was used to study the correlation between soil P fractions and soil properties (*p* < 0.05 and *p* < 0.01).

## 5. Conclusions

The ecological restoration in the alpine sandy lands of the eastern edge of the Tibetan Plateau can increase the content of TP and TPi while reducing the content of TPo. Ecological restoration measures can improve the content of soil labile inorganic P (resin-P, NaHCO_3_-Pi, and NaHCO_3_-Po) by activating NaOH-Pi and HCl-P, thereby improving the availability of soil P and increasing the potential P source residual-P, and the effect is better in MG and SG. SOC and TN have a significant relationship with soil TP and the content of each fraction and is an important factor affecting the content of soil P. It is worth noting that under different ecological restoration modes, significant distribution differences were shown among soil layers, and the change range of the 20–40 cm soil layer in MG mode and the 40–60 cm soil layer in SG mode was greater than that in other soil layers under the same mode. Therefore, the MG and SG models have significant effects on the accumulation and availability improvement of soil P in alpine sandy land, making them relatively suitable restoration models. The findings of this study contribute to understanding the availability and potential P sources of soil under ecological restoration, providing a scientific basis for selecting ecological restoration modes and enhancing the effectiveness and efficiency of restoration in alpine sandy areas.

## Figures and Tables

**Table 1:** Soil total phosphorus (TP), total inorganic phosphorus (TPi), and total organic phosphorus (TPo) content characteristics under different ecological restoration modes.

Depth (cm)	Treatment	TPi (mg·kg^−1^)	TPo (mg·kg^−1^)	TP (g·kg^−1^)
0–20	CK	74.97 ± 6.40 ^b^	33.17 ± 10.37 ^b^	0.11 ± 0.00 ^b^
	MG	64.91 ± 9.66 ^b^	59.89 ± 12.16 ^a^	0.12 ± 0.02 ^a^
	SA	91.02 ± 7.11 ^a^	35.61 ± 7.92 ^b^	0.13 ± 0.00 ^a^
	SG	72.03 ± 7.30 ^b^	53.53 ± 5.19 ^a^	0.13 ± 0.01 ^a^
20–40	CK	78.55 ± 4.01 ^a^	25.33 ± 5.38 ^c^	0.10 ± 0.01 ^c^
	MG	57.93 ± 10.86 ^b^	107.84 ± 13.10 ^a^	0.17 ± 0.02 ^a^
	SA	79.94 ± 9.94 ^a^	50.19 ± 15.20 ^b^	0.13 ± 0.02 ^b^
	SG	69.54 ± 4.71 ^ab^	53.82 ± 8.66 ^b^	0.12 ± 0.01 ^bc^
40–60	CK	84.37 ± 6.69 ^a^	20.50 ± 5.58 ^c^	0.10 ± 0.01 ^c^
	MG	59.19 ± 9.12 ^b^	63.28 ± 13.41 ^b^	0.12 ± 0.01 ^b^
	SA	78.50 ± 5.50 ^a^	54.55 ± 9.19 ^b^	0.13 ± 0.01 ^b^
	SG	54.92 ± 7.18 ^b^	137.07 ± 20.76 ^a^	0.19 ± 0.01 ^a^

Note: Different letters above each bar indicate significant differences among different restoration years at *p* < 0.05. Vertical bars denote the standard error of the means (*n* = 4).

**Table 2:** Relationship between soil phosphorus content and basic physical and chemical properties.

Index	TP	TPi	TPo	Resin-P	NaHCO_3_-Pi	NaHCO_3_-Po	NaOH-Pi	NaOH-Po	HCl-P	Residual-P
SWC	0.444 **	−0.289 *	0.455 **	0.090	0.084	0.337 *	0.193	0.497 **	−0.503 **	0.231
BD	−0.386 **	0.590 **	−0.522 **	−0.184	−0.152	−0.584 **	0.401 **	−0.457 **	0.503 **	−0.385 **
pH	−0.460 **	0.176	−0.426 **	0.116	−0.121	−0.295 *	−0.043	−0.377 **	0.250	−0.400 **
SOC	0.733 **	−0.639 **	0.812 **	0.457 **	0.420 **	0.633 **	−0.469 **	0.702 **	−0.593 **	0.761 **
TN	0.794 **	−0.726 **	0.892 **	0.504 **	0.410 **	0.762 **	−0.508 **	0.751 **	−0.678 **	0.841 **

Note: Values in the table are Pearson’s correlation coefficients; * indicates significant correlation at the 5% level (*p* < 0.05); ** indicates significant correlation at the 1% level (*p* < 0.01).

**Table 3:** Basic information on plant communities under different restoration modes.

Treatment	Elevation (m)	Slop (°)	Height (m)	Crown Breat (m)	Coverage (%)
CK	3420	<5	-	-	<5
MG	3416	<5	-	-	94.3 ± 0.8
SA	3422	<5	1.52 ± 0.11	1.90 ± 0.13	6.6 ± 1.4
SG	3417	<5	1.64 ± 0.15	1.91 ± 0.14	81.4 ± 8.9

## Data Availability

The original contributions presented in this study are included in the article. Further inquiries can be directed to the corresponding author.
